# Strategies for Reducing Blood Transfusions in Hepatic Resection

**DOI:** 10.1155/1994/98027

**Published:** 1994

**Authors:** T. Matsumata, H. Itasaka, K. Shirabe, M. Shimada, K. Yanaga, K. Sugimachi

**Affiliations:** Second Department of Surgery Faculty of Medicine Kyushu University Fukuoka Japan

## Abstract

A comparison of 60 blood transfused and 71 nonblood transfused hepatic resection patients was done to evaluate strategies for reducing blood transfusions during hepatic surgery. There were no significant differences between the two groups with regard to preoperative laboratory data, except for prothrombin time and hematocrit value. The mean operative blood loss was 1990 ml and 760 ml in the blood transfused and nonblood transfused groups, respectively. A multivariate analysis suggested that the patient’s body weight, preoperative prothrombin time, and operative blood loss independently predicted the need for intraoperative blood transfusion. Major postoperative complications developed more frequently in the blood transfused group than in the nonblood transfused group (31.7 vs. 11.3%, *p*<0.005). These results suggest that the difference in operative blood loss between the two groups was related to the prolonged prothrombin time and a susceptibility for blood transfusion was found to exist particularly in patients with a lower hematocrit value as well as a lower body weight. Thus, the improvement of these preoperative laboratory data combined with avoiding the use of the hematocrit value as a determining factor for intraoperative transfusion could correspond to a reduction in operative blood loss, while curtailing the demands on blood bank facilities, and lowering the risk of postoperative complications.


*HPB Surgery*, 1994, Vol. 8, pp. 1–7
Reprints available directly from the publisher
Photocopying permitted by license only

© 1994 Harwood Academic Publishers GmbH
Printed in Malaysia

### Strategies for Reducing Blood Transfusions in Hepatic Resection

T. MATSUMATA, H. ITASAKA, K. SHIRABE, M. SHIMADA, K. YANAGA, and K. SUGIMACHI

Second Department of Surgery, Faculty of Medicine, Kyushu University, Fukuoka, Japan

A comparison of 60 blood transfused and 71 nonblood transfused hepatic resection patients was done to evaluate strategies for reducing blood transfusions during hepatic surgery. There were no significant differences between the two groups with regard to preoperative laboratory data, except for prothrombin time and hematocrit value. The mean operative blood loss was 1990 ml and 760 ml in the blood transfused and nonblood transfused groups, respectively. A multivariate analysis suggested that the patient's body weight, preoperative prothrombin time, and operative blood loss independently predicted the need for intraoperative blood transfusion. Major postoperative complications developed more frequently in the blood transfused group than in the nonblood transfused group (31.7 vs. 11.3%, $p < 0.005$). These results suggest that the difference in operative blood loss between the two groups was related to the prolonged prothrombin time and a susceptibility for blood transfusion was found to exist particularly in patients with a lower hematocrit value as well as a lower body weight. Thus, the improvement of these preoperative laboratory data combined with avoiding the use of the hematocrit value as a determining factor for intraoperative transfusion could correspond to a reduction in operative blood loss, while curtailing the demands on blood bank facilities, and lowering the risk of postoperative complications.

KEY WORDS: Hepatocellular carcinoma hepatic resection blood transfusion ultrasonic dissector prothrombin time

### INTRODUCTION

The liver constiutes a huge friable vascular sponge with a great capacity for bleeding. Many previous reports have shown a correlation between intraoperative blood loss and the postoperative clinical course[1–4]. During hepatectomy interruption of the portal pedicle at the hilus, Pringle's maneuver[5] or a vascular clamp[6] placed at the hilus, prior to transection of the parenchyma, is useful in minimizing blood loss. Surgeons have used techniques such as the "control method"[7] or the finger fracture technique[8] for the control of hemorrhaging during hepatic resection.

In an effort to better control blood loss from the cut surface of the liver, the ultrasonic dissector has been proposed as a useful technical aid in liver resection[9,10]. However, the use of this device does not eliminate the problem of blood loss from the myriad of capillaries, particularly if a coagulation defect exists.

We have used on ultrasonic dissector since April 1985, and the mean estimated blood loss during hepatic surgery was significantly reduced from 2510 ml in the earlier period (1981–1985) to 1317 ml more recently (1985–1988)[11]. However, intraoperative red blood cell transfusions were performed in about half of the patients in the recent period. We describe here our results, of 131 hepatic resections performed between 1985 and 1990 for hepatocellular carcinoma, to clarify the limitations of this new device, and to discuss the disadvantages of blood transfusion in hepatic surgery.

### PATIENTS AND METHODS

131 consecutive Japanese patients with hepatocellular carcinoma who had undergone hepatic resection at the Second Department of Surgery, Kyushu University

Hospital between April 1985 and December 1989 were the subjects for the present study. The ages of the patients ranged from 33 to 76 years, with an average of 57.4 years. There were 111 men and 20 women. Eighty-one of the 131 patients (62%) had associated liver cirrhosis. In all patients the abdomen was explored through a bilateral subcostal incision with a para-xyphoid extension, the abdominal incision was extended to the thoracic area in one patient. Hemihepatectomies was performed in 17 (two extended right hepatectomies, 10 right hepatectomies, three left hepatectomies, and two central bisegmentectomies), segmentectomies in 27, subsegmentectomies in 35, and partial hepatectomy less than a subsegment in 52 patients.

The standard hepatectomy was performed by utilizing our vascular clamping method at the hepatic hilus[12]. A left lateral segmentectomy was done using a dissection and interruption of the portal pedicle in the umbilical fissure[13]. In the other hepatic resections vascular control at the hepatic hilus was obtained with either the Pringle maneuver[5], (maximum duration of liver ischemia being 15 minutes) in continuity or a modification of Pringle's maneuver[14] or the hemihepatic vascular control method[6] with the maximal duration of liver ischemia being 30 minutes in continuity.

All patients were hospitalized for at least two weeks following hepatic resection. Major complications occurred insome, particularly those patients with chronic liver disease.

The patients were divided into two groups. The blood transfusion (BT) group patients received intraoperative red blood cell replacement and the non-blood transfusion (NBT) group patients were managed without any blood replacement intraoperatively. The hematocrit levels were measured intraoperatively every hour, and a red blood cell transfusion was started whenever the hematocrit value decreased to $31.8 \pm 6.2\%$. One patient in the BT group was given a red blood cell transfusion preoperatively, and post-operative red blood cell replacements were given in 12 and two patients respectively of the BT and the NBT groups.

We compared the clinical and laboratory data, perioperative management, and the post-operative courses of the two groups.

A Macintosh SPSS statistical package (SPSS Inc., Chicago, Illinois, U.S.A.) was used for the analysis. Student's t test and the chi-square test were performed to compare the groups. A logistic regression analysis with a forward stepwise procedure was employed to identify predictive factors for intraoperative blood transfusion. *P* values of less than 0.05 were considered to be significant. All values are expressed as the mean ± standard deviation.

### RESULTS

There were 60 and 71 patients in the BT and the NBT groups, respectively. Table 1 demonstrates that there was no significant difference with respect to the clinical data and preoperative laboratory data between the two groups except for the prothrombin time and hematocrit value.

There were 15 (25%) and eight patients (11%) with hematocrit values of less than 35% in the BT and the NBT groups, respectively ($p < 0.05$). There was a significant difference between the male (14/111, 13%) and female (7/20, 35%) patients with hematocrit values of less than 35% ($p < 0.025$), including two with microcytic anemia and 11 with macrocytic anemia. One female patient with a preoperative hematocrit of 27.8% underwent a red blood cell transfusion during hepatic resection in spite of only 400 ml estimated blood loss during surgery.

The operations in both groups were performed by the same surgical team. There was no difference in distribution among the types of liver resection between the two groups. A hepatic hilar dissection for preresectional control of the vascular structures was performed in 42 and 51 patients in the BT and the NBT groups, respectively. Except for the hepatic resections, in which there is no ischemic insult to the remnant liver by vascular clamping at the hepatic hilus, the duration of remnant liver ischemia was not significantly different ($58.7 \pm 38.3$ vs. $43.7 \pm 25.8$ minutes) between the 31 BT group patients and the 32 NBT group patients who underwent hepatic resection using vascular clamping. The use of equipment, including an ultrasonic dissector was essentially the same in the two groups (Table 2).

The mean operation time was 333 minutes and 298 minutes ($p < 0.05$), and the mean operative blood loss was 1990 ml and 760 ml ($p < 0.001$) in the BT and NBT groups, respectively. The mean volume of red blood cell replacement for the BT group was 1160 ml, which corresponded to that of a 60% operative blood loss. The mean tumor size was 4.3 cm and 2.9 cm in the BT and the NBT groups, respectively ($p < 0.01$), however, there was no significant difference between the two groups with respect to the weight of the resected liver (Table 3).

**Table 1** Clinical and preoperative laboratory data.

| *Parameters* | *Blood Transfusion* ($n = 60$) | *Nonblood Transfusion* ($n = 71$) |
|---|---|---|
| Age (yr) | 57.7 ± 8.7 | 57.1 ± 9.5 |
| Sex (male:female) | 48:12 | 63:8 |
| Height (cm) | 161.1 ± 8.6 | 160.0 ± 6.9 |
| Weight (kg) | 57.5 ± 9.2 | 56.9 ± 8.3 |
| Accompanying changes in liver parenchyma | | |
| Cirrhosis | 40(67%) | 41(58%) |
| Fibrosis | 8(13%) | 4(6%) |
| Chronic hepatitis | 12(20%) | 22(31%) |
| Fatty change | 0 | 2(3%) |
| Previous upper abdominal surgery | 17(28%) | 14(20%) |
| Associated conditions | | |
| Esophageal varices | 20(33%) | 19(27%) |
| Diabetes mellitus | 21(35%) | 20(28%) |
| Peptic ulcer | 2(3%) | 10(14%) |
| Cholelithiasis | 8(13%) | 2(3%) |
| Ischemic heart disease | 3(5%) | 3(4%) |
| Obstructive lung disease | 4(7%) | 3(4%) |
| Preoperative laboratory data | | |
| Serum albumin (3.5 – 5.0 g/dl)[a] | 3.66 ± 0.37 | 3.74 ± 0.48 |
| Bilirubin (0.2 – 1.2 mg/dl) | 0.75 ± 0.31 | 0.74 ± 0.31 |
| SGOT (0 – 40 U/L) | 59.6 ± 25.7 | 58.7 ± 28.7 |
| SGPT (0–40 U/L) | 76.9 ± 46.1 | 83.9 ± 45.7 |
| Indocyanine green test (0–10%) | 16.3 ± 9.9 | 16.9 ± 9.6 |
| Prothrombin time[b] (80–100%) | 82.1 ± 17.9 | 88.7 ± 12.9 |
| Fibrinogen (150–360 mg/dl) | 260 ± 114 | 267 ± 118 |
| Hemoglobin (12–18 g/dl) | 12.6 ± 1.7 | 13.1 ± 1.4 |
| Hematocrit[c] (35–52%) | 37.9 ± 4.9 | 39.6 ± 4.0 |
| Platelet count (14–44 × 10 mm) | 13.1 ± 6.4 | 14.3 ± 7.0 |

SGOT = serum glutamic oxaloacetic transaminase; SGPT = serum glutamic pyruvic transaminase; [a] normal ranges are given in parentheses; [b] $p < 0.01$; [c] $p < 0.05$.

**Table 2** Types of liver resection and operative methods.

| *Parameters* | *Blood Transfusion* ($n = 60$) | *Nonblood Transfusion* ($n = 71$) |
|---|---|---|
| Types of liver resection | | |
| Extended right hepatectomy | 1 | 1 |
| Right hepatectomy | 3 | 7 |
| Left hepatectomy | 0 | 3 |
| Central bisegmentectomy | 1 | 1 |
| Left lateral segmentectomy | 6 | 7 |
| Other segmentectomies | 10 | 4 |
| Subsegmentectomy | 18 | 17 |
| Partial hepatectomy | 21 | 31 |
| Operative methods | | |
| Hepatic hilar dissection | | |
| Yes | 42(31)[a] | 51(32)[a] |
| No | 18 | 20 |
| Intraoperative aids | | |
| Ultrasonography | 60 | 71 |
| Liver clamps | 3 | 1 |
| Ultrasonic dissector | 44 | 59 |
| Microwave tissue coagulator | 31 | 26 |

[a] the number in parenthesis indicates patients who underwent hepatic resection, with vascular clamping at the hepatic hilus.

**Table 3** Intraoperative Risk Factors and Management.

| *Parameters* | *Blood Transfusion* ($n = 60$) | *Nonblood Transfusion*($n = 71$) |
|---|---|---|
| Operation time[a] (min) | 333 ± 87 | 298 ± 105 |
| Intraoperative | | |
| blood loss[b] (ml) | 1990 ± 990 | 760 ± 400 |
| water replacement (ml) | 3530 ± 1220 | 3240 ± 1080 |
| albumin replacement[c] (g) | 18.4 ± 16.7 | 10.2 ± 14.3 |
| urine output (ml) | 692 ± 397 | 669 ± 360 |
| Tumor size[c] (cm) | 4.3 ± 3.3 | 2.9 ± 1.8 |
| Weight of resected liver (g) | 230 ± 249 | 219 ± 283 |

[a] $p < 0.05$; [b] $p < 0.001$; [c] $p < 0.01$.

**Table 6** Morbidity and Mortality.

| *Parameters* | *Blood Transfusion* ($n = 60$) | *Nonblood Transfusion* ($n = 71$) |
|---|---|---|
| Complications[a] | 19(31.7%) | 8(11.3%) |
| Intraabdominal bleeding | 2 | 1 |
| Bile leakage | 5 | 2 |
| Intraabdominal abscess[b] | 7 | 2 |
| Persistent hyperbilirubinemia | 0 | 1 |
| Upper gastrointestinal bleeding | 1 | 1 |
| Uncontrollable ascites | 6 | 6 |
| Operative deaths | 0 | 0 |
| Hospital deaths | 1 | 1 |
| Hospital stay after surgery[c] (days) | 34.1 ± 15.3 | 26.6 ± 10.6 |

[a] $p < 0.005$; difference between the two groups; [b] $p < 0.05$; [c] $p < 0.01$.

**Table 4** Risk factors for intraoperative blood transfusion: Stepwise logistic regression analysis.

| *Factors* | *Coefficient* | *Odds ratio* | *P value* |
|---|---|---|---|
| Body weight | −0.113 | 0.876 | 0.0017 |
| Prothrombin time | 0.831 | 2.294 | 0.0036 |
| Blood loss | 0.004 | 1.004 | 0.0000 |

The significant risk factors predicting the blood transfusion were body weight, preoperative prothrombin time, and intraoperative blood loss (Table 4).

Serial changes in laboratory data after hepatic resection are shown in Table 5. In the BT group, lactic dehydrogenase levels on the 1st, 3rd, and 7th postoperative days were significantly higher, compared to those in the NBT group ($p < 0.01$, on the 1st and the 3rd; $p < 0.001$, on the 7th postoperative days). There were no significant differences between the groups with respect to the value of direct bilirubin, although, the indirect bilirubin level on the 1st postoperative day in the BT group was significantly higher than that in the NBT group ($p < 0.01$). The minimum levels for hemoglobin and hematocrit values were over 10 g/dl and 30%, respectively, and these were noted on the 3rd postoperative day.

Major postoperative complications developed in 19 (31.7%) and eight (11.3%) patients of the BT and NBT groups, respectively ($p < 0.005$). The rate of intra-abdominal abscess in the BT group was more frequent than that in the NBT group ($p < 0.05$). Persistent hyperbilirubinemia in the NBT group was encountered after hemostasis for intra-abdominal bleeding following right hepatic lobectomy. In addition, the length of hospital stay of patients after surgery in the NBT group was significantly shorter than that in the BT group ($p < 0.01$) (Table 6).

**Table 5** Serial Changes of Laboratory Data after Hepatic Resection.

| *Parameters* | *Transfusion* | *Postoperative days* 1 | 3 | 7 |
|---|---|---|---|---|
| SGPT (U/L) | Yes | 341 ± 311[a] | 289 ± 233[b] | 115 ± 70[a] |
| | No | 253 ± 151 | 197 ± 126 | 95 ± 45 |
| Lactic dehydrogenase | Yes | 534 ± 299[b] | 312 ± 82[b] | 280 ± 78[c] |
| (U/L) | No | 419 ± 157 | 267 ± 75 | 240 ± 48 |
| Direct bilirubin | Yes | 0.55 ± 0.44 | 0.46 ± 0.24 | 0.35 ± 0.43 |
| (mg/dl) | No | 0.44 ± 0.30 | 0.44 ± 0.43 | 0.34 ± 0.41 |
| Indirect bilirubin | Yes | 1.23 ± 0.44[b] | 0.99 ± 0.44 | 0.71 ± 0.35 |
| (mg/dl) | No | 1.00 ± 0.54 | 0.84 ± 0.53 | 0.64 ± 0.43 |
| Hemoglobin (g/dl) | Yes | 12.2 ± 1.8 | 10.4 ± 1.7 | 11.3 ± 1.6 |
| | No | 12.1 ± 1.5 | 10.3 ± 1.6 | 11.0 ± 1.8 |
| Hematocrit (%) | Yes | 36.5 ± 5.3 | 31.3 ± 5.2 | 34.0 ± 5.0 |
| | No | 36.7 ± 4.6 | 30.9 ± 4.8 | 32.8 ± 5.2 |

SGPT = serum glutamic pyruvic transaminase; Significantly different from the nonblood Transfusion group, at [a]$p < 0.05$, [b]$p < 0.01$, and [c]$p < 0.001$, respectively.

### DISCUSSION

This study shows that control of the vascular structures at the hepatic hilus before resection combined with the use of an ultrasonic dissector can lead to a reduction in intraoperative blood loss of up to approximately 700 ml, even for cirrhotic livers if coagulation is normal. However, parenchymal diseases of the liver are usually associated with a decreased synthesis of plasma-clotting proteins resulting frequent coagulation disorders. Depressed levels of prothrombin in liver disease are partly due to subclinical vitamin K deficiency[15] and/or a latent consumption coagulopathy[16], and once any underlying bleeding tendencies are identified, timely vitamin K treatment and/or heparin treatment can substantially improve blood coagulation[17,18].

The hematological study in our 21 patients with hematocrit values of less than 35% might suggest that the 11 patients with macrocytic anemia. which is common in chronic liver disease,[18] might have benefited from preoperative folate therapy. Preoperative iron treatment for some patients may also remove the need for blood transfusion.

We performed a red blood cell transfusion whenever the hematocrit value reached 31.8 ± 6.2%, to maintain hematocrit levels higher than 30%. The mean volume of red blood cell replacement for the BT group amounted to 60% of intraoperative blood loss, this maintained the hematocrit values at over 30% postoperatively. Such hematocrit values, however, might not necessarily be optimal for patients with good cardiac function undergoing hepatic resection, because myocardial infraction was found to be less than one fourth as common in cirrhotic patients as in noncirrhotic patients[19]. Even high-risk patients with coronary disease easily tolerate postoperative hematocrits of 25 to 30%[20,21]. It is apparent that patients with good cardiac function are able to tolerate a greater degree of anemia[22,23]. In addition to this, Babineau *et al.*[24] reported that red blood cell transfusions based on a hemoglobin value of less than 10 g/dl did not significantly affect oxygen consumption in surgical intensive care patients. The hematocrit or hemoglobin level thus should not be a trigger for intraoperative transfusion, particularly in women with relatively low hematocrit values[25], or with a relatively low body weight.

Although the distribution among the types of the hepatic resections and the weight of the resected livers were essentially the same between the two groups, major postoperative complications developed more frequently in the BT group than in the NBT group.

The postoperative lactic dehydrogenase levels in the BT group were significantly higher compared to those in the NBT group. In addition, although there was no significant difference between the groups with respect to the value of direct bilirubin, the indirect bilirubin level on the 1st postoperative day in the BT group was significantly higher than that in the NBT group ($p < 0.01$). These results suggest that hemolysis of the transfused red blood cells occurred in patients undergoing hepatic resection. Ostrow *et al.*[26] showed that a discrete episode of hemolysis results in jaundice within twenty-four hours. Splenic sequestration of the red blood cells can depress the splenic clearance function and increase the susceptibility to septic challenge[27]. Jensen *et al.*[28] also described that the natural killer cell function was significantly impaired up to 30 days after surgery in patients transfused with whole blood.

Although the conditions requiring blood transfusion might be the true underlying cause of postoperative complications[29], blood transfusion however seems to increase risk factors, particularly for postoperative infections in the current study, as well as in those reported previously by others[30,31]. Hence, as Jamieson *et al.*[1] described, we believe it is worthwhile carrying out hepatic resections without giving blood transfusions as far as possible, to avoid the possible short-term deleterious effects of perioperative blood transfusions. In addition, because the association between transfusion and recurrence-free survival after hepatic resection was recognized in patients with early stage hepatocellular carcinoma without intrahepatic metastasis[32], we must also avoid unnecessary blood transfusion in hepatic surgery for hepatocellular carcinoma in order to obtain the best prognosis.

In conclusion, this study verified that the preresectional control of the vascular structures at the hepatic hilus combined with the use of an ultrasonic dissector can lead to a reduction of intraoperative blood loss of up to approximately 700 ml, even for cirrhotic livers if blood coagulation is normal.

The difference in operative blood loss between the BT and NBT groups was related to the prolonged prothrombin time. There is a tendency to transfusion patients with a lower hematocrit value. Since 1990 it has been our policy to use hemodynamic signs instead of hematocrit levels as a transfusion trigger, and the present hematocrit values used to indicate the start of intraoperative transfusion are now between 20 and 25%.

### REFERENCES

1. Jamieson, G. G., Corbel, L., Campion, J. P., and Launois, B. (1992) Major liver resection without a blood transfusion: is it a realistic objective? *Surgery*, **112**, 32–36.
2. Delva, E., Camus, Y., Nordlinger, B., Hannoun, L., Parc, R., Deriaz H., Lienhart, A., and Huguet, C. (1989) Vascular occlusions for liver resections: operative management and tolerance to hepatic ischemia: 142 cases. *Ann. Surg.*, **209**, 211–218.
3. Ekberg, H., Tranberg, K. G., Andersson, R., Jeppsson, B., and Bengmark, S. (1986) Major liver resection: perioperative course and management. *Surgery*, **100**, 1–8.
4. Pommier, R. F., Woltering, E. A., Campbell, J. R., and Fletcher, W. S. (1987) Hepatic resection for primary and secondary neoplasms of the liver. *Am. J. Surg.*, **153**, 428–433.
5. Pringle, J. H. (1908) Notes on the arrest of hepatic hemorrhage due to trauma. *Ann. Surg.*, **48**, 541–549.
6. Makuuchi, M., Mori, T., Gunven, P., Yamazaki, S., and Hasegawa, H. (1987) Safety of hemihepatic vascular occlusion during resection of the liver. *Surg. Gynecol. Obstet.*, **164**, 155–158.
7. Pack, G. T., and Baker, H. W. (1953) Total right hepatic lobectomy: report of a case. *Ann. Surg.*, **188**, 253–258.
8. Lin, T. Y., Chen, K. M., and Liu, T. K. (1960) Total right hepatic lobectomy for primary hepatoma. *Surgery*, **48**, 1048–1060.
9. Andrus, C. H., and Kaminski, D. L. (1986) Segmental hepatic resection utilizing the ultrasonic dissector. *Arch. Surg.*, **121**, 515–521.
10. Hodgson, W. J. B., Morgan, J., Byrne, D., and DelGuercio, L. R. M. (1992) Hepatic resections for primary and metastatic tumors using the ultrasonic surgical dissector. *Am. J. Surg.*, **163**, 246–250.
11. Matsumata, T., Kanematsu, T., Shirabe, K., Sonoda, T., Furuta, T., and Sugimachi, K. (1990) Decreased morbidity and mortality rates in surgical patients with hepatocellular carcinoma. *Br. J. Surg.*, **77**, 677–680.
12. Matsumata, T., Kanematsu, T., Yamagata, T., Utsunomiya, K., Yanaga, K., and Sugimachi, K. (1992) Simplified hilar division in controlled right hepatectomy. *Am. J. Surg.*, **163**, 339.
13. Matsumata, T., Kanematsu, T., Shirabe, K., Yamagata, M., Utsunomiya, T., and Sugimachi, K. (1992) Controlled left lateral segmentectomy of the liver. *Hepato-gastroenterol.*, **39**, 177–181.
14. Matsumata, T., Kanematsu, T., Shirabe, K., Yamagata, M., Utsunomiya, T., Furuta, T., and Sugimachi, K. (1991) Modified technique of Pringle's maneuver in resection of the liver. *Surg. Gynecol. Obstet.*, **172**, 245–246.
15. Liebman, H. A., Furie, B. C., and Furie, B. (1982) Hepatic vitamin K-dependent carboxylation of blood-clotting proteins. *Hepatology*, **2**, 488–494.
16. Cordova, C., Musca, A., Violi, F., Alessandri, C., and Vezza, E. (1982) Improvement of some blood coagulation factors in cirrhotic patients: treated with low doses of heparin. *Scand. J. Haematol.*, **29**, 235–240.
17. MacIntosh, E. L., and Minuk, G. Y. (1992) Hepatic resection in patients with cirrhosis and hepatocellular carcinoma. *Surg. Gynecol. Obstet.*, **174**, 245–254.
18. Kimber, C., Deller, D. J., Ibbotson, R. N., and Lander, H. (1964) The mechanism of anaemia in chronic liver disease. *Quarterly Journal of Medicine*, **34**, 33–64.
19. Vanecek, R. (1976) Atherosclerosis and cirrhosis of the liver. *Bull. World Health Organ.*, **53**, 567–570.
20. Verska, J. J., Ludington, L. G., and Brewer, L. A. III (1974) A comparative study of cardiopulmonary bypass with nonblood and blood prime. *Ann. Thorac. Surg.*, **18**, 72–80.
21. Bayer, W. L., Coenen, W. M., Jenkins, D. C., and Zucker, M. L. (1980) The use of blood and blood components in 1,769 patients undergoing open-heart surgery. *Ann. Thorac. Surg.*, **29**, 117–122.
22. Czer, L. S. C., and Shoemaker, W. C. (1978) Optimal hematocrit value in critically ill postoperative patients. *Surg. Gynecol. Obstet.*, **147**, 363–368.
23. Spence, R. K., Carson, J. A., Poses, R., McCoy, S., Pello, M., Alexander, J., Popovich, J., Norcross, E., and Camishion, R. C. (1990) Elective surgery without transfusion: influence of preoperative hemoglobin level and blood loss on mortality. *Am. J. Surg.*, **159**, 320–323.
24. Babineau, T. J., Dzik, W. H., Borlase, B. C., Baxter, J. K., Bistrian, B. R., and Benotti, P. N. (1992) Reevaluation of current transfusion practices in patients in surgical intensive care units. *Am. J. Surg.*, **164**, 22–25.
25. Friedman, B. A., Burns, T. L., and Schork, M. A. (1980) An analysis of blood transfusion of surgical patients by sex: a quest for the transfusion trigger. *Transfusion*, **20**, 179–188.
26. Ostrow, J. D., Jandl, J. H., and Schmid, S. (1962) The formation of bilirubin from hemoglobin in vivo. *J. Clin. Invest.*, **41**, 1628–1636.
27. Grover, G. J., and Loegering, D. J. (1982) Effect of splenic sequestration of erythrocytes on splenic clearance function and susceptibility to septic peritonitis. *Infect. Immun.*, **36**, 96–102.
28. Jensen, L. S., Andersen, A. J., Christiansen, P. M., Hokland, P., Juhl, C. O., Madsen, G., Mortensen, J., Moller-Nielsen, C., Hanberg-Sorensen, F., and Hokland, M. (1992) Postoperative infection and natural killer cell function following blood transfusion in patients undergoing elective colorectal surgery. *Br. J. Surg.*, **78**, 513–516.
29. Yanaga, K., Kanematsu, T., Takenaka, K., and Sugimachi, K. (1986) Intraperitoneal septic complications after hepatectomy. *Ann. Surg.*, **203**, 148–152.
30. Tartter, P. I. (1988) Blood transfusion and infectious complications following colorectal cancer surgery. *Br. J. Surg.*, **75**, 789–792.
31. Ford, C. D., VanMoorleghem, G., and Menlove, R. L. (1993) Blood transfusions and postoperative wound infection. *Surgery*, **113**, 603–607.
32. Matsumata, T., Ikeda, Y., Hayashi, H., Kamakura, T., Taketomi, A., and Sugimachi, K. (1993) The association between transfusion and cancer-free survival after curative resection for hepatocellular carcinoma. *Cancer*, **72**, 1866–1871.

### INVITED COMMENTARY

The article written by T. Matsumata *et al.* addresses the most important issue of bloodless liver surgery. Prevention of hemorrhage during liver resection has dramatically improved in the last two decades and the aim at the end of this century is the performance of all kinds of hepatic surgery without any blood transfusion.

The reasons to avoid blood loss and consequently blood transfusion are multiple: cost, risk of infectious diseases (hepatitis, CMV, AIDS), immunodepression with early (increased morbidity) and late (increased risk of malignant recurrency) consequences.

Progress in surgical strategy has allowed us to realize more and more frequently "bloodless" liver resections. The technical means to prevent and reduce hemorrhage during liver transection include vascular clamping, (either Pringle maneuver or total vascular isolation), Kellyclasy, ultrasonic dissector, fibrin glue application on the raw surface of the liver, argon beam coagulator.... Combination of several techniques may be beneficial and result in increased safety and low operative mortality and morbidity.

Another means to reduce the incidence of blood transfusion in liver surgery is to revise the traditional criteria of blood replacement. Preoperative anemia is not exceptional in chronic liver diseases and is most often well tolerated. The classical strategy of mathematical replacement of per operative blood loss is obsolete. In several fields of surgery, blood replacement has appeared unnecessary when hemoglobin was 8 g/dl or higher, without any hemodynamic nor metabolic deleterious effect. Spontaneous correction of anemia occurs in the first post operative weeks. It could also be possible in some cases to organize auto transfusion with prior collection of the patients own blood.

The paper of Dr. Matsumata illustrates the changing strategy in the use of blood replacement in the last few years. To keep hematocrit values over 30% is certainly xcessive and leads to over transfusion with potential adverse effects. Restrictive criteria to administer blood infusion are beneficial in terms of economy, prevention of infections diseases and tumor biology. In any case, precise surgical technique of liver resection is the prerequisite for a smooth uncomplicated post operative course since it has been established that morbidity and mortality are related to intra-operative hemorrhage.

To reduce blood transfusions in hepatic resection supposes the combined action of the surgeon and the anesthesiologist. The surgeon should minimize blood loss. The anesthesiologist should reconsider the rationale of blood replacement which is not always necessary.

P. C. Huguet
Department of Surgery
Princess Grace Hospital
MONACO, Principality of MONACO

